# Disordered eating in autistic trans and gender diverse people: a lived experience-led scoping review

**DOI:** 10.1186/s40337-025-01447-z

**Published:** 2025-11-19

**Authors:** Luka C. J. White, Kai Schweizer, Kai S. Thomas

**Affiliations:** 1 Department of Health and Social Sciences, University of Edinburgh, Edinburgh, Scotland, UK; 2 School of Human Sciences, University of Western Australia, Perth, Australia; 3 School of Psychology, Cardiff University, Cardiff, Wales, UK

**Keywords:** Transgender, Autistic, Eating disorders, Disordered eating behaviours

## Abstract

**Background:**

This lived experience-led scoping review explores the evidence base related to eating disorders/disordered eating behaviours in Autistic trans and gender diverse (TGD) people. This review highlights the currently available data on eating disorder prevalence rates, comparisons with allistic and cisgender groups, drivers and maintenance factors, the relationship between eating disorders and gender-affirming medical care, and treatment outcomes in this population.

**Methods:**

We conducted a search of the databases ProQuest, Medline, CINAHL, Cochrane Library, Web of Science, Scopus, and PsycINFO for articles relating to eating disorders/disordered eating behaviours in Autistic TGD people. Five articles published between 2021 and 2025 met the criteria for the review.

**Results:**

The included articles were predominantly cross-sectional studies (*n* = 4) and one case series (*n* = 1). Researchers used a mixture of self-reported and clinically recorded eating disorder diagnoses, as well as validated measures, including the Eating Disorder Examination Questionnaire and the Nine-Item Avoidant/Restrictive Food Intake Disorder Scale. The literature highlights that the prevalence of eating disorders/disordered eating behaviours is high in the Autistic TGD population, and suggests that atypical presentations may be particularly common. Possible drivers and maintenance factors include sensory hypersensitivity, co-occurring Attention-Deficit Hyperactivity Disorder, gender dysphoria and passing concerns, and appearance pressures idealising thinness. Limited data were available on the role of gender-affirming medical care or eating disorder treatments, or on treatment outcomes.

**Discussion:**

Further research is needed to better understand the nuances of eating disorders/disordered eating behaviours in Autistic TGD people. Key to future research inquiries should be the adoption of an intersectional approach, co-production of research with Autistic TGD people, and research considering treatment outcomes.

## Abstract (plain language summary)

We reviewed existing literature on eating disorders/disordered eating behaviours (EDs/DEBs) in Autistic trans and gender diverse (TGD) people. Five studies met our criteria. All were high-quality pieces published in the last five years. We found that Autistic TGD people have particularly high rates of EDs/DEBs and that atypical presentations may be more common. Autistic TGD people are exposed to the same risk factors for EDs/DEBs as the general population, which interact with TGD- and Autism-specific risk factors, co-occurring neurodivergence, and sensory processing differences. Evidence about treatment outcomes and the impact of gender-affirming medical care is limited. Given the additional and unique risks for EDs/DEBs in Autistic TGD people, more research is needed in this emerging field.

## Introduction

There is increasing evidence demonstrating that eating disorders (EDs) and disordered eating behaviours (DEBs) are particularly prevalent in trans and gender diverse (TGD) and Autistic populations [1–4]. TGD is an umbrella descriptor for people whose gender is incongruent with their sex presumed at birth, including trans men, trans women, nonbinary, agender and genderqueer people, and is also used here to include those with traditional or Indigenous genders that exist outside of Western definitions of gender, such as Two-Spirit people or Faʻafāfine [5]. However, we are mindful that language is constantly evolving, and TGD may not be the preferred term for all. Non-TGD people are described as cisgender. Neurodivergent is an umbrella term for those whose minds ‘*function in ways which diverge significantly from the dominant societal standards of “normal*”’ [6]. Non-neurodivergent people are described as neurotypical. Autistic describes a type of neurodivergence (a neurotype). Autistic people share a pattern of thinking, moving, sensing and communicating which is different from neurotypical standards of normal. The impact of these differences on the daily lives of Autistic people varies widely, with people reporting different levels of disability through these differences not being accommodated for in neurotypical-centred (neuronormative) societies. Non-Autistic people are described as allistic. This review uses identity-first language and capitalises the word Autistic in line with recommendations from Autistic advocates [7]. EDs are mental health conditions characterised by severe difficulties with eating or eating-related behaviour which may be associated with impairments in physical health and psychosocial functioning [8]. According to the Diagnostic and Statistical Manual, 5th Edition (DSM-5), these conditions can be clinically diagnosed as anorexia nervosa (AN), bulimia nervosa (BN), binge-eating disorder (BED), avoidant-restrictive food intake disorder (ARFID), and other specified feeding or eating disorder (OSFED) [8]. Atypical EDs, including atypical AN and other EDs that fall under OSFED, make up the largest proportion of EDs globally, followed by BED and then BN [9, 10]. EDs can be life-threatening, with AN and BN associated with increased mortality, including death by suicide, compared to the general population and those with other mental health conditions [11–13]. DEBs are potentially harmful patterns of eating or eating-related behaviour, present in EDs, but which also commonly occur at a subclinical level in the general population [14].

TGD people consistently report elevated levels of ED/DEB compared to cisgender comparison groups [2, 15], with between 20 and 50% engaging in DEB, and approximately 30% screening positive for ED symptoms [4]. These prevalence rates may be underestimates, as psychometric measures to screen for ED/DEB were initially validated in cisgender samples and may not reflect the best measurement tools for TGD populations [16]. Between 2 and 12% of TGD people have an ED diagnosis [4], whilst the lifetime risk for developing an ED is estimated at 8.4% for cisgender women and 2.2% for cisgender men [9]. Trans men show elevated scores on psychometric measures such as the Eating Disorder Examination Questionnaire (EDE-Q) compared to trans women, cisgender women, and cisgender men, while trans women show higher scores compared to cisgender men [2, 15]. Studies suggest nonbinary people show even higher scores and a higher lifetime risk of developing an ED, compared to trans men [17, 18]. TGD people are more likely to present to ED treatment services at younger ages and with more anxiety and depression symptoms compared to their cisgender counterparts [19, 20]. In addition, TGD people with EDs may be more at risk of self-injurious and suicidal thoughts and behaviours than those without EDs or cisgender people with EDs [21, 22]. For example, in one sample, 74.8% of TGD people with a diagnosed ED reported a suicide attempt in the past year [22]. TGD people, along with sexual minorities, are at risk of misdiagnosis with Borderline Personality Disorder (BPD) due to these high rates of self-harm and suicidal ideation [23–25]. Whilst BPD and ED diagnoses commonly overlap [26], the concept of BPD has a complex and problematic history and misdiagnosis with BPD can cause significant harm for TGD people [27]. These findings underscore the need for research to better understand the mechanisms driving EDs in this underserved and marginalised group.

Being TGD is not an inherent risk factor for EDs, rather, TGD people experience common risk factors for ED development and maintenance to cisgender people and in addition, they experience unique risk factors related to their TGD identity and societal cisgenderism. For example, gender dysphoria may drive TGD people to engage in DEBs as a way of suppressing features socially attributed to their presumed sex at birth or accentuating features socially attributed to their gender [28–35]. Examples include trans men engaging in DEBs to lose weight and suppress their menses or flatten their chests, or trans women engaging in DEBs to lose weight because thinness is socially attributed to femininity. Gender-affirming medical care, such as hormone replacement therapy, can decrease ED symptoms in TGD people who experience gender dysphoria by facilitating physical changes to the body that align with their gender [2, 15, 28, 30, 32, 36]. This effect may be more pronounced for trans men than trans women, with one study finding that the risk of an ED diagnosis increased for trans women following hormone replacement therapy [37]. Loria and colleagues [37] hypothesise that one explanation for this is that trans women on hormone therapy are at an increased risk of developing restrictive eating to pass as cisgender and thus achieve safety, in a culture that promotes the thin ideal for women. Gender minority stress, including discrimination, violence and rejection related to a minority gender identity, has been associated with ED/DEB in TGD populations in cross-sectional studies [38–40]. Internalised transphobia emerged as a significant proximal minority stressor in longitudinal analysis, suggesting this may be particularly important in the maintenance of ED symptoms over time [41]. Another factor in ED/DEB maintenance may be that TGD people often report experiencing negative interactions with healthcare professionals and concealing their TGD identity in ED treatment settings [31, 36, 42]. Therefore, TGD people may experience unique factors driving both the development and maintenance of their ED.

Neurodivergence and gender diversity overlap as part of natural human variation; an example of this is that at least 11% of TGD people are Autistic [43]. Autistic people may eat differently from allistic people. For example, preferring to eat familiar foods or avoiding certain foods due to sensory aversions [44]. However, as well as Autistic eating differences, Autistic people also screen higher on measures of ED symptoms compared to allistic comparison groups [45, 46]. This is particularly the case for measures of ARFID symptomology, with as many as 21% of Autistic people screening positive for ARFID symptoms, and a recent meta-analysis finding an 11.41% prevalence of ARFID diagnoses in Autistic groups, compared to 0.3–15.5% of the general population [47–49]. The prevalence of AN, BN and BED ranges from 1.4 to 7.9% in Autistic people [1, 3]. These may be underestimated, particularly the rates of BN and BED, due to the limited literature on non-restrictive EDs for Autistic people, and the use of psychometric measures which were validated in neurotypical populations.

Being Autistic has been framed as a risk factor for ED/DEB, with ample evidence showing that certain Autistic traits are associated with ED/DEB [50]. This includes cognitive traits (such as a greater preference for predictability and routine, a tendency towards more literal interpretation, a greater intensity of interests, and more executive functioning difficulties), emotional traits (such as differences in identifying and communicating their emotions) and sensory traits (such as differences in interoception and in the intensity of their bodily response to sensory stimuli) [50–52]. However, not all Autistic people go on to develop an ED and EDs are well known to be influenced by environmental factors external to the individual. Research into environmental factors predicting the development and maintenance of EDs in Autistic people is in its infancy; however, data from qualitative research points to the impact of bullying, abuse, and societal intolerance of difference leading to Autistic people feeling different and defective from a young age [51, 53]. For some Autistic people, restrictive eating becomes a way to cope with these difficult emotions or is an attempt to fit in with neurotypical peers [51, 53]. Autistic people may also mask their Autistic traits in an attempt to fit in, and this camouflaging has also been linked to DEBs [54]. Autistic minority stress is predictive of poor well-being, depression, anxiety, stress, and post-traumatic stress [55, 56]. These mental health concerns co-occur with ED/DEBs [57], however, there is currently no research investigating the role of Autistic minority stress in predicting ED/DEB. Another factor influencing the maintenance of ED/DEBs in Autistic people may be that Autistic people describe being seen as uncooperative or too complex by ED services, to the extent that at times they are refused treatment [58–60]. These experiences of accessing ED services may exacerbate ED symptoms and lead to delayed access to treatment, resulting in complex and severe ED presentations.

Despite many TGD people being Autistic, little is known about the prevalence of ED/DEBs and their underlying mechanisms in Autistic TGD people. Autistic TGD people are reported to experience high rates of depression, anxiety, post-traumatic stress, and suicidality, which highly co-occur with ED/DEBs, compared to Autistic or TGD groups alone [56, 57, 61, 62]. Given this trend, and as rates of ED/DEB are high in both Autistic and TGD groups, it is plausible that Autistic TGD people are particularly at risk of ED/DEBs. It is similarly plausible that Autistic TGD people will be exposed to both TGD-specific and Autism-specific risk factors, as well as unique intersectional experiences of ED/DEB, treatment and recovery. This may result in Autistic TGD people having more severe or complex presentations of ED/DEB than their allistic and/or cisgender peers.

This scoping review aims to map the current literature on ED/DEB in Autistic TGD people. Specifically, this review aims to: (1) synthesise and compile the existing research on ED/DEB in Autistic TGD people and (2) assess the methodological quality of the included literature. A scoping review approach was chosen due to the limited existing research and lack of previous reviews related to this topic.

## Methods

### Positionality

All co-authors are gender diverse, and some also hold intersecting identities as Autistic and or having lived experience of an eating disorder. We hold professional roles in clinical, research, and advocacy settings related to neurodivergence, gender diversity, and eating disorders across multiple countries. We are also white, live in high-income countries, and are not intellectually disabled, and recognise that our experiences cannot speak to all the intersecting experiences of the wider Autistic TGD community. We adopt a neurodiversity-affirming framework in which Autism and other forms of neurodivergence are understood as natural human variation rather than pathology. In this paper, that commitment shapes both language and interpretation. We purposefully use non-pathologising and non-stigmatising terms for neurodivergence, such as traits, features, and characteristics, and avoid stigmatising language such as disorder, abnormal, or impairment. We use community preferred language, including person-first language where appropriate and capitalisation of the A in Autism. We also adopt a gender-affirming framework that recognises trans and gender diverse identities as valid expressions of human diversity, rather than conditions requiring correction. This commitment likewise shapes our language and interpretation. We use inclusive, non-pathologising terms such as trans, nonbinary, and gender diverse, and avoid outdated or stigmatising language such as transgendered, biologically male or female, or gender identity disorder. We follow people’s self-described names and pronouns, and centre lived experience rather than presumed sex at birth. This framework guided how we assessed and discussed the evidence, including how cisnormative assumptions and barriers to gender affirming care can create or intensify eating disorder risk and persistence. We further recognise that concepts of neurodivergence, gender, and eating disorders have been shaped largely by Western clinical traditions, and we acknowledge how this may influence interpretation and applicability across cultures and contexts. We name these positions to be transparent about how our social locations and expertise informed the interpretation and presentation of this review.

### Inclusion criteria

This review included peer-reviewed studies published in English that described EDs/DEBs in Autistic TGD people. To be eligible, studies had to include participants whose gender identity was incongruent with their sex presumed at birth and who had an ED diagnosis or DEBs. Studies were only eligible if participants self-identified or were diagnosed as Autistic, or where Autistic traits were measured in the sample of TGD people. Studies that included cisgender participants were only eligible if they provided stratified data for TGD participants. All study designs are eligible for inclusion, including quantitative, qualitative, mixed methods, case reports, and case series. Quantitative studies needed to either describe self-reported EDs or DEBs or use validated tools such as the EDE-Q to report an ED diagnosis or ED symptoms. Qualitative studies were only eligible if themes and participant quotes could clearly be attributed to Autistic TGD people with EDs/DEBs. Studies were excluded if they focused exclusively on cisgender or allistic participants or examined eating behaviours without a specific focus on Autistic TGD people. The following were excluded from this study: book chapters, review articles, editorials, clinical opinion pieces, non-English language studies, and dissertations.

### Search strategy

**Fig. 1:**
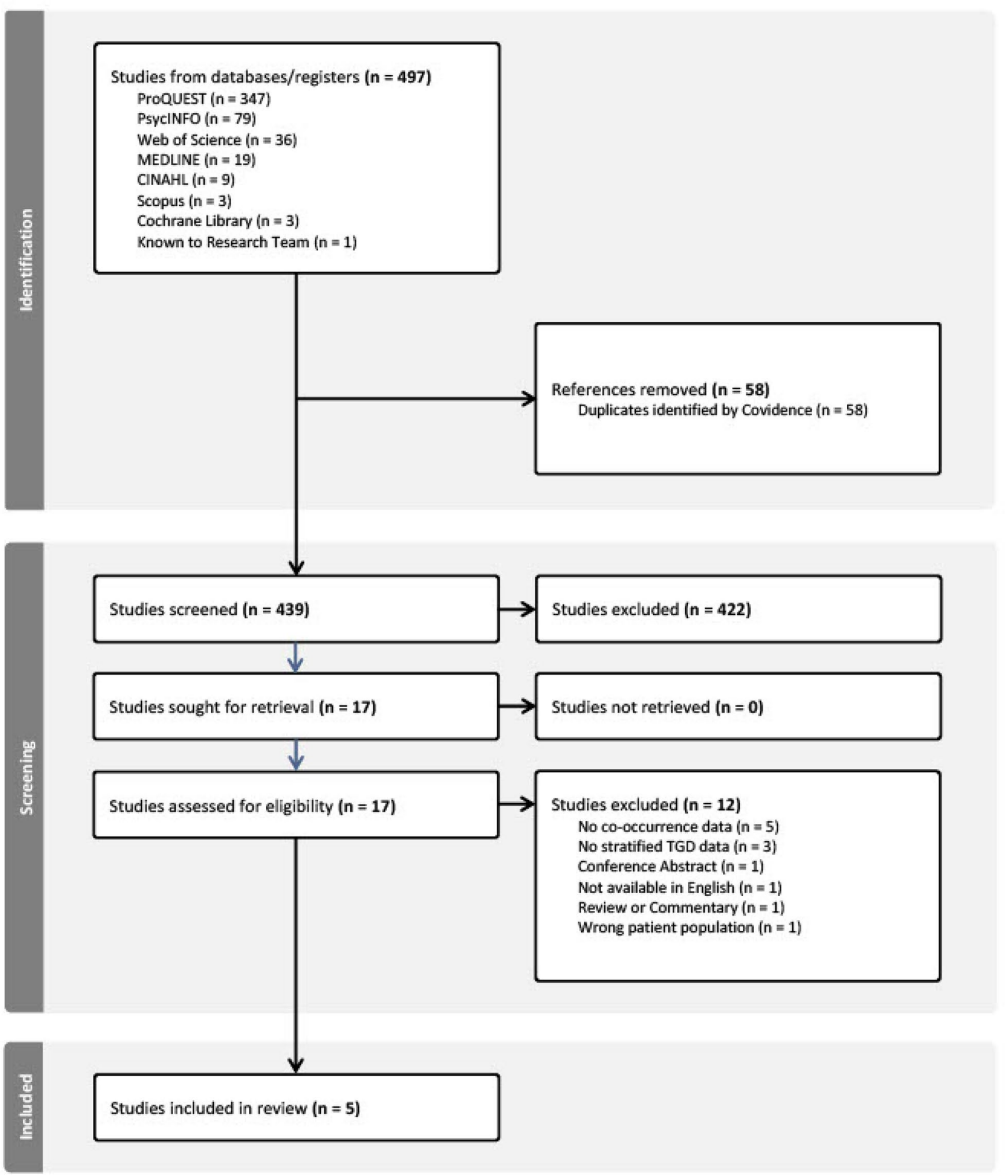
Search strategy

The search strategy was developed through initial meetings between the first and second author. ​​A database search was conducted using ProQuest, Medline, CINAHL, Cochrane Library, Web of Science, Scopus, and PsycINFO. The search string comprised three key categories: (1) ED/DEB terms (“eating disorder” OR “eating disorders” OR “anorexia nervosa” OR bulimi* OR “binge eating” OR binge-eating OR “purging disorder” OR “binge eating disorder” OR “Avoidant restrictive food intake disorder” OR “other specified feeding and eating disorder” OR pica OR “rumination disorder” OR “night eating syndrome” OR “food intake disorder” OR orthorexia OR “disordered eating” OR Dieting OR fasting OR overeating OR “binge eating” OR “self-induced vomiting” OR purging OR “driven over-exercise” OR “compulsive exercise” OR laxative OR diuretic OR “diet pill” OR “non-prescription steroid use” OR “chewing and spitting” OR “chew and spit” OR “body checking”); (2) Autism-related terms (ASD OR ASC OR autism OR “autism spectrum disorder” OR “autism spectrum condition” OR “pervasive developmental delay”), and (3) gender identity terms (transgender* OR transsexual* OR “gender identity” OR “gender dysphoria” OR “gender dysphoric” OR dysphoric OR “Gender diverse” OR “gender diversity” OR genderfluid OR non-binary OR sistergirl OR brotherboy OR two-spirit OR “gender identity disorder” OR “gender incongruence”). No limits were set by date of publication. The initial search was conducted in December 2024. A second search was conducted in April 2025. A detailed breakdown of the search strategy is provided in Fig. 1.

The first and second authors conducted data extraction and charting. Relevant details for each article, including the year of publication, country, study design, gender identity of participants, Autism status, data collection methods, and key findings related to the co-occurrence of ED/DEBs, Autism, and gender diversity were recorded.

Methodological quality was independently assessed by the first and second authors using the tools and guidelines developed by Kmet and colleagues [63]. This framework employs a 10-item checklist for qualitative studies and a 14-item checklist for quantitative studies, assessing methodological quality based on predefined criteria. Each item was scored as ‘yes’ = 2, ‘partial’ = 1, ‘no’ = 0, or N/A when applicable. The methodological quality score for each study was calculated by dividing the total score by the total possible score, generating a value between 0.0 and 1.0, where higher scores indicate stronger methodological quality. Any discrepancies in quality assessment scores were resolved through discussion between the reviewers, with a third reviewer (KT) consulted to mediate and establish the final methodological quality assessment scores. A summary of these assessments is presented in Table 1.

**Table 1:** Quality score of included studies

	Score
Kahn et al., 2023	1.00
Pham et al., 2021	1.00
Sedgewick et al., 2021	0.77
Strauss et al., 2021	1.00
Thomas et al., 2025	1.00

## Results

### Article characteristics

All articles included were published between 2021 and 2025. Of the five included articles, four were quantitative cross-sectional studies [46, 64–66] and one was a case series [67]. Two of the studies were conducted in the United States [64, 67], one in Australia [65], and two in the United Kingdom [46, 66]. Three articles explicitly included non-binary participants [46, 65, 66], while the remaining two either included only binary trans men and trans women or did not specify non-binary inclusion [64, 67]. Among the cross-sectional studies, one compared Autistic TGD people to allistic TGD people [65], two compared Autistic and allistic TGD participants to Autistic and allistic cisgender participants [46, 64], and one measured Autistic traits in an exclusively TGD sample [66]. To determine Autistic status, three studies used self-reported Autism diagnoses [46, 65, 66] while two relied on clinical records [64, 67], Additionally, one study provided Autism severity specifiers (i.e., level 1, level 2) and Social Responsiveness Scores [67], and another two provided Autism Quotient (AQ) scores alongside self-reported diagnoses [46, 66]. The included study that examined Autistic traits in a TGD sample found that 88.5% scored above the screening cutoff score (> 65) for a probable autism diagnosis on the AQ-short, with the mean score in the sample at 80.02 [66]. This study interpreted the AQ-short in line with the cut-off suggested by Hoekstra and colleagues [68]. The study that examined Autistic traits in self-reported Autistic sample found that the mean score on the AQ-short was 21.03, above the proposed cutoff of 19 [46]. This study used an abbreviated version of the AQ-short, hence the difference in cutoff scores. Two studies included participants under the age of 18 only [64, 67], and one study included participants aged 14–25 [65]. Participants’ ages across all five articles ranged from 9 to 81.29 years. Race and ethnicity data were reported in three articles [46, 64, 66] with the most common descriptors being White, Mixed or multiple, Hispanic or Latinx, Black, and Asian.

Details on gender-affirming medical care were provided in two articles [65, 67]. One article specified the medical transition of participants [67], whilst the other provided data on social transition, “partial” social transition, medical transition, and whether participants wanted but were unable to access medical transition [65].

The included studies utilised a range of methods to assess EDs/DEBs. One study relied solely on self-reported ED diagnoses [65] one collected detailed data on DEBs, body mass index (BMI), BMI percentage of estimated target weight, and DEXA scan results for bone mineral density and body fat percentage [67], and one determined ED diagnoses via clinical records [64]. Two included articles utilised a validated measure, assessing ED symptoms via the EDE-Q [46, 66] or the Nine-Item ARFID screen (NIAS) [66].

### Quality assessment

Included articles were assessed using the Checklist for Assessing the Quality of Quantitative Studies [63] with scores ranging from 0.77 to 1 (see Table 1).

### Prevalence of eating disorders and disordered eating behaviours

Two articles reported the prevalence of EDs in their samples of Autistic TGD people [64, 65]. Utilising clinical records, Kahn and colleagues [64] reported a prevalence of 10.1%. Whereas, using self-reported diagnoses, Strauss and colleagues [65] reported a prevalence of 52.3%. One study reported the levels of ED symptoms captured using the EDE-Q and found that Autistic TGD people had a mean score of 1.88, below the clinical cut-off of 2.8 [46]. A fourth study [66] reported levels of shape and weight concern on the EDE-Q in TGD people with high Autistic traits, and found that the sample had mean scores of 3.54 in the shape concern subscale and 3.12 in the weight concern subscale, below the clinical cutoff of 4. These findings were broadly consistent across gender identities, with trans feminine people reporting the highest mean scores (shape concern, M = 3.66, weight concern M = 3.31), followed by trans masculine people (shape concern, M = 3.65, weight concern M = 3.24), then nonbinary people, (shape concern M = 3.54, weight concern M = 3.05) with gender expansive people having the lowest mean scores (shape concern, M = 3.16, weight concern, M = 2.77). This study also measured ARFID symptoms captured using the NIAS and found that, in the sample of TGD people with elevated Autistic traits, the mean score was 15.91, below the clinical cut off of 23 [66]. This study found that 29.1% of participants scored above the NIAS picky eating subscale cutoff, 26.9% scored above the NIAS appetite cutoff, and 12.6% scored above the NIAS-fear cutoff. ARFID symptoms (NIAS total score) were highest in the gender expansive group (M = 19.11) and trans feminine group (M = 17.14) and lowest in the trans masculine (M = 14.97) and nonbinary (M = 14.88) groups. Specific ED diagnoses and DEBs in Autistic TGD people were reported by one study [67]. This case series described reported diagnoses of OSFED, ARFID, and Unspecified Feeding or Eating Disorder (UFED) in their three cases. One article reported ED diagnoses in the Autistic and allistic groups, not separated by gender, and found that the Autistic group were more likely to report AN, BN, BED, OSFED and ARFID diagnoses, with the rate of OSFED being particularly elevated compared to the allistic group (6.8% vs. 1.9%) [46]. A further article reported ED diagnoses in a TGD sample, not separated into Autistic and allistic groups, and found that 15.4% of the sample reported an ED diagnosis, with AN being the most common, followed in order by EDNOS, ARFID and BN [66]. Pham and colleagues described a range of DEBs in their case series of Autistic TGD youth, including calorie counting, restricting intake, eliminating high-fat and high-sugar foods, skipping meals, eating infrequently, binge eating, and eating a limited variety of foods [67].

### Comparisons between groups

#### Autistic vs. allistic TGD people

Autistic TGD people were found to have a higher lifetime prevalence of an ED diagnosis than allistic TGD people across two studies (52.3% vs. 14.2%, 10.1% vs. 8.4%) [64, 65]. The average predicted probability of an ED diagnosis in Autistic TGD people was 0.05 (CI 0.03–0.07) compared to 0.04 (CI 0.03–0.05) in allistic TGD people [64]. In Strauss and colleagues’ work, the difference in ED diagnoses between Autistic TGD (*n* = 172) and allistic TGD (*n* = 687) groups was statistically significant [65]. The difference in Kahn and colleagues’ work was not statistically significant (Autistic TGD *n* = 464, allistic TGD *n* = 4,925). A further study found that Autistic TGD (*n* = 143) people reported a marginally higher average EDE-Q scores than allistic TGD (*n* = 18) people (M = 1.88 vs. 1.70) [46]. However, whether this difference was statistically significant was not reported. This study found that, regardless of their gender, Autistic groups were significantly more likely to have been diagnosed with an ED and to report ED symptoms than allistic groups.

#### TGD vs. cisgender autistic people

Two studies provided data comparing TGD and cisgender Autistic people’s eating pathology [46, 64]. Autistic TGD people (*n* = 464) were found to have a higher lifetime prevalence of ED diagnosis than Autistic cisgender (*n* = 40,249) people (10.1% vs. 4.3%) [64]. However, this difference was not found to be statistically significant. The average predicted probability of an ED diagnosis in Autistic TGD people was 0.05 (CI 0.03–0.07) compared to 0.04 (CI 0.04–0.06) in Autistic cisgender people [64]. In another study, Autistic TGD people had a marginally higher average score on the EDE-Q (*n* = 143, M = 1.88) than the Autistic cisgender male group (*n* = 71, M = 1.80) but lower than the Autistic cisgender female group (*n* = 317, M = 2.30) [46]. Whether these differences were statistically significant was not reported.

#### Autistic TGD vs. allistic cisgender people

Autistic TGD people were found to have a higher lifetime prevalence of ED diagnosis than allistic cisgender people (10.1% vs. 1.1%), with an odds ratio of 5.97 (CI: 3.94–9.05) [64]. The average predicted probability of an ED diagnosis in Autistic TGD people was 0.05 (CI 0.03–0.07) compared to 0.01 (CI 0.01–0.01) in the allistic cisgender group [64]. In adults, Autistic TGD people report a higher average score (*n* = 143, M = 1.88) than allistic cisgender males (*n* = 54, M = 1.38) but lower than allistic cisgender females (*n* = 327, M = 2.01) [46]. Whether this was a statistically significant difference was not reported.

### Interaction effects

The impact of Autistic identity and gender identity on levels of ED symptoms was explored in one study [46]. Sedgewick and colleagues conducted a series of robust multiple regressions to test the effects of gender and Autism status on EDE-Q total scores [46]. Autism status was entered first, followed by gender, and an interaction effect was tested. When all participants were included, Autistic groups were significantly more likely to report ED symptoms than allistic groups, regardless of gender. Irrespective of whether they were Autistic, cisgender women were significantly more likely to report ED symptoms than cisgender men. However, there was no significant difference between cisgender women and the TGD group or between cisgender men and the TGD group. Although Autism status and gender independently contributed to the level of ED symptoms reported, the interaction effect was not significant. Therefore, the effects of gender and Autism status did not significantly depend on each other to impact levels of ED symptoms. When the regressions were run with participants with an ED diagnosis excluded, no significant difference was found between Autistic and allistic participants, or between cisgender and TGD groups, and no interaction effect was found.

### Drivers and maintenance factors

One study qualitatively described the drivers and maintenance factors contributing to DEBs in Autistic TGD people. Pham and colleagues [67] presented three case studies of Autistic TGD people with EDs, each with unique contributing factors to their ED presentation. In two of the young people included, gender dysphoria and passing concerns were key to their DEBs and motivations for weight loss. One of these young people, a transmasculine person, described intentional weight loss as a strategy to minimise their curves and appear more masculine. He also described concern about increased appetite following testosterone treatment, which led to him engaging in binge eating. Similarly, a transfeminine person initially attributed her restriction to executive functioning difficulties and low motivation, but later acknowledged engaging in intentional weight loss to appear more feminine, associating thinness with femininity. In contrast, the third young person denied weight or shape concerns. Instead, their weight loss was due to restriction triggered by sensory sensitivity to food texture and smell, alongside parental restriction of sensorily tolerable ‘junk food’.

A further study examined the role of Autistic traits, Attention-Deficit Hyperactivity Disorder (ADHD) traits, and sensory sensitivities in predicting ARFID symptoms in a TGD sample. Thomas and colleagues [66] found significant positive correlations between Autistic traits and ARFID symptoms (*r* =.405, *p* <.001), Autistic traits and sensory sensitivities (hypersensitivity, *r* =.629, *p* <.001, hyposensitivity, *r* =.399, *p* <.001), and sensory sensitivities and ARFID symptoms (hypersensitivity, *r* =.557, *p* <.001, hyposensitivity, *r* =.403, *p* <.001). A moderate association between Autistic traits and ARFID symptoms was retained when EDE-Q shape and weight concerns were controlled for in a correlational analysis, *r* =.389, *p* <.001. This study included a measure of ADHD traits and found that in the sample of TGD adults with high Autistic traits, ADHD traits were weakly positively correlated with ARFID symptoms (*r* =.185, *p* <.05). A multiple linear regression analysis investigated the predictive effect of Autistic traits, ADHD traits and sensory sensitivities on ARFID symptoms whilst controlling for sex assigned at birth and weight and shape concerns. The final model was significant, *F* = 7.521, *p* <.001, R^*2*^ = 0.329, however sensory hypersensitivity remained the only significant unique predictor (β *=* 0.499, *p* <.001). The authors report that an exploratory analysis confirmed a similar pattern of data when weight and shape concerns were not controlled for in the model.

### Access to gender-affirming medical care

One study compared whether Autistic TGD people had accessed or could access gender-affirming medical care to allistic TGD people [65]. This found that Autistic TGD people were more likely to want gender-affirming hormones and surgery but not be able to access them and were less likely overall to have accessed such intervention, compared to allistic TGD people. However, this study did not investigate the effect of these differences on ED diagnoses or DEBs. The case series of three Autistic TGD youth found that in two cases, DEBs persisted following access to gender-affirming hormone therapy, with one individual finding that whilst testosterone therapy improved their mood and suicidal ideation, it also increased their appetite and weight and they began to engage in binge eating [67].

### Treatment outcomes

The case series described the treatment types offered to three Autistic TGD youth [67]. All were provided with gender-affirming medical care and an Autism diagnosis, if they did not already have one. ED treatments included dietitian input, an ED specialist therapist, a dialectical therapist, and pharmacological treatment. All three were described to improve following treatment with regards to reduced restriction and weight gain, however, DEBs were described to continue in two cases. In the transmasculine young person, binge eating persisted, and in a transfeminine young person, whilst initially improvements were sustained, they had begun restrictive eating again by the final appointment recorded.

## Discussion

### Prevalence

ED/DEBs in Autistic TGD people is a recently emerging area of research. This review demonstrates that Autistic TGD people may have particularly high rates of ED/DEBs. However, there remains a significant variability in the exact prevalence estimates. This may be in part due to the discrepancy between collected self-reported ED diagnoses and EDs on clinical records, an issue previously highlighted in the wider TGD population [15]. EDs may also be underdiagnosed in TGD populations, meaning the prevalence of self-reported ED symptoms may be higher than self-reported ED diagnoses [69]. Further indicators that the prevalence of ED/DEBs may be higher than estimated include that most of the studies in this review relied on clinical records or self-reported Autism diagnosis for inclusion, likely missing the large number of Autistic people who do not have an Autism diagnosis [70]. Future research could include undiagnosed Autistic people by using trait-based approaches, which can further be used to quantitatively examine relationships between Autistic traits and ED symptoms in the general population (see [71]). Research is also increasingly including self-identified Autistic people, recognising that such individuals are experts in their own experience who face multiple barriers to diagnosis [72, 73]. For example, the diagnostic criteria for Autism in the DSM-5 [8] were primarily developed based on research and clinical observations of cisgender males, which has important implications for how Autism is diagnosed and understood, particularly among TNB people. This can result in Autistic TNB people being misdiagnosed with mental health conditions over Autism [74]. Prevalence studies should also include Autistic TGD individuals from across the age spectrum, as three of the five studies included here focus on those under the age of 25, mirroring a trend in wider ED/DEB research [75].

This review presents preliminary evidence that Autistic TGD people may experience ED presentations that differ from conventional diagnostic criteria. This is in line with research suggesting higher rates of OSFED, atypical AN and ARFID in TGD people [20, 76] and elevated ARFID prevalence in Autistic people [47–49]. Within this context, this review highlights a pattern of ARFID symptomology in TGD people with high Autistic traits. Current ED diagnostic frameworks and screening tools were developed primarily for cisgender, allistic populations, and may therefore not fully capture the experiences of Autistic TGD people. Atypical presentations in Autistic TGD people suggest that the use of traditional screening tools, such as the EDE-Q, are insufficiently nuanced to understand ED presentations in Autistic TGD people. Future research should disaggregate findings by specific ED/DEBs presentations to best understand whether specific types of ED symptoms (such as restrictive vs. binge/purge type) may be driven by different mechanisms in Autistic TGD people compared to cisgender Autistic or allistic TGD people. This is particularly pertinent as binge-type presentations and ARFID presentations are understudied in the wider ED research field [10, 77].

### Drivers and maintenance factors

Currently, very limited data exists on drivers and maintenance factors for ED/DEBs in Autistic TGD people, with only two studies relevant to the review exploring the mechanisms underlying EDs in this group. Potential mechanisms include a combination of gender dysphoria and passing concerns, sensory sensitivities, and appearance pressures idealising thinness. The sociocultural context shapes appearance pressures, which typically promote thinness as the ideal for cisgender women and muscularity as the ideal for cisgender men, which leads to a subset of individuals engaging in DEBs to achieve these body shapes [78, 79]. Autistic people may be particularly vulnerable in this regard, due to attempting to ‘fit in’ with their neurotypical peers, or differences in social information processing and literal thinking styles [51, 80]. Further exploration is needed to determine whether Autistic TGD people internalise appearance pressures for their presumed sex at birth, for their gender identity, or both. Recent evidence suggests that TGD people presumed male at birth may be particularly at risk of DEBs relating to a drive for thinness, whereas people presumed female at birth may be more at risk of those relating to a drive for muscularity [71], therefore internalising appearance pressures in line with their gender identity. This review supports the preliminary assumption that sensory hypersensitivity is an important driver of ARFID symptomology in Autistic TGD groups. Being Autistic is associated with sensory processing differences [81], similarly being ADHD [82], and sensory processing differences are also overrepresented in the wider TGD population [83]. Further evidence is required to understand whether sensory hypersensitivity contributes to EDs/DEBs in Autistic TGD populations beyond ARFID, and to understand the nuanced contributions of Autistic traits, ADHD traits and sensory processing differences. The lack of statistical interaction found between Autism status and gender in Sedgewick and colleagues’ paper [46] does not erase the likelihood of compounded intersectional marginalisation contributing to Autistic TGD people developing EDs/DEBs. As Schweizer and colleagues [84] report, being both neurodivergent and TGD may create unique eating experiences, even if these do not meet the criteria for DEBs or ED symptoms.

There was minimal data considering the impact of overlapping neurodivergence (such as co-occurring ADHD) despite evidence that being Autistic and ADHD frequently overlap [85], and that ADHD is itself associated with a higher prevalence of EDs [86, 87]. This review found that in one sample of TGD adults with high Autistic traits, ADHD traits were associated with ARFID symptoms [66], leading to a preliminary hypothesis that the prevalence of EDs in Autistic TGD adults may be partially contributed to by co-occurring ADHD. This novel association between ADHD traits and ARFID symptoms in TGD adults highlights the importance of considering co-occurring Autistic and ADHD traits in future ED research with Autistic TGD populations. There was also a lack of data considering the impact of intersecting identities beyond autism and gender, such as ethnicity, sexual orientation and socio-economic status. Early evidence suggests that having multiple minority identities equates to a greater risk of EDs [88, 89]. This is consistent with findings for depression and anxiety in Autistic TGD populations who experience dual minority status, with a compounded greater risk for those that are also sexual minorities [61]. Intersectionality theory provides a useful lens for understanding ED risk in multiply marginalised groups [90]. Future studies with Autistic TGD populations could utilise such an approach to examine the structural and systemic factors that intersect to contribute to ED/DEBs, as well as to examine unique opportunities for resilience, coping and survivorship.

### Treatment gaps

A key evidence gap in this area is the absence of any meaningful data on treatment outcomes for Autistic TGD people, with only one study relevant to this review exploring this topic and finding that symptoms persisted in two out of three cases despite ED treatment. This is particularly important for future research, given the evidence that Autistic traits and TGD identity predict poorer long-term psychological outcomes for individuals with EDs [91, 92]. Compounded negative experiences in ED treatment settings may contribute to poorer long-term outcomes for Autistic TGD people, as both Autistic and TGD groups separately report negative experiences in ED treatment settings [31, 36, 42, 58–60]. Recent qualitative findings record that neurodivergent TGD people with EDs experience difficulty having their intersectional experience acknowledged and accommodated [84]. This review provides preliminary evidence that gender-affirming medical care may be important for some Autistic TGD individuals with EDs, whilst being less likely than allistic peers to have access to such intervention. This is important to note, as Autistic TGD individuals are at times denied gender-affirming medical care due to tropes that Autistic people cannot understand themselves and make healthcare decisions [93, 94]. This is despite evidence that Autistic TGD people have stable understandings of their gender over time, no evidence of increased transition-related regret or detransition in Autistic TGD people, and decision-making capacity being individually determined rather than diagnosis-specific [94, 95]. This review also suggests that Autistic TGD people may be particularly likely to want gender-affirming medical care, in line with some evidence that Autistic TGD people may have particularly strong gender dysphoria [96], and that gender dysphoria may be exacerbated by Autistic sensory hypersensitivity [97, 98].

### Strengths and limitations

This work represents a comprehensive scoping review of the available literature on Autistic TGD EDs/DEBs. We are TGD authors, some of whom are Autistic and/or have experience of an ED. Whilst this no doubt influences our reflexivity, we feel that our lived experience comprises a significant strength, given calls from mental health and disability groups that such work should not be done ‘about us, without us’. A key limitation of this review is the small number of papers included. Whilst reflective of the newly emergent nature of this field of study, in practice, this means we can only draw tentative conclusions about the prevalence rates of EDs/DEBs in Autistic TGD people and the drivers and maintenance factors for EDs/DEBs. Furthermore, conclusions regarding treatment outcomes are not possible given the lack of available literature on this topic, except for a single case series. The strength of our findings is also directly linked to the strength of the available evidence, which, whilst largely of high quality, may not have included fully representative samples. For example, there was a lack of inclusion of self-identified Autistic people and, in some cases, nonbinary people. Three of the five studies focused on young people. Sample ethnicity, socioeconomic status, and disability were rarely reported, and all studies came from the United States, the United Kingdom, or Australia. Reflecting trends in the broader literature, there was a lack of representation of BN and BED in the reviewed studies. Further, there was heterogeneity in the ways that EDs/DEBs were measured in the reviewed papers, with two articles relying on the 28-item version of the EDE-Q. This has not, to our knowledge, been validated in Autistic or TGD samples, and may conflate ED concepts with common Autistic needs and behaviours [99]. Autistic identity was similarly captured in varying ways, including self-reported diagnosis, clinical records and trait measures. We restricted our search to English-language articles and excluded dissertations and grey literature, which may have additionally limited the findings of this review.

### Future directions

We recommend that future research should focus on understanding how clinicians can best support Autistic TGD people, as well as a nuanced understanding of resilience and recovery in Autistic TGD groups. ED screening and treatment tools should be adapted to accommodate the sensory, communicative and gender-related needs of the Autistic TGD population. Longitudinal studies could examine the temporal relationships between neurodivergence, gender diversity and the development of EDs, this may be particularly pertinent when considering the impact of gender-affirming medical care on ED symptoms in Autistic TGD groups. Research into Autistic EDs and TGD EDs should specifically include Autistic TGD people in their samples. Clinical practice with Autistic TGD people must embrace gender-affirming and neuro-affirming approaches, such as those outlined by Cobbaert and colleagues [100] and Chang and colleagues [101].

Finally, none of the papers included in this review specifically discussed whether Autistic TGD people were involved in the co-production of these studies. This is despite calls for research into Autistic TGD mental health to be led by Autistic TGD people [102], a call echoed in research with Autistic people with EDs [103] and TGD people with EDs [104]. Future research into Autistic TGD ED/DEBs should be co-designed and co-produced with Autistic TGD people, to ensure that it is in line with community priorities.

## Conclusion

Research on EDs/DEBs in Autistic TGD people is still an emergent field, with only five studies published in the last five years. The existent literature was considered to be of high overall methodological quality, but methodologically heterogeneous. Current findings suggest that the prevalence of EDs/DEBs may be elevated in Autistic TGD people, though more consistent measurements are needed to better understand whether this prevalence is higher compared to TGD allistic or Autistic cisgender groups. Despite limited data, the authors cautiously suggest that atypical ED presentations may be particularly common in Autistic TGD populations. Drivers of EDs/DEBs in Autistic TGD populations include those affecting the general population, which intersect with Autism-specific and TGD-specific stressors, co-occurring additional forms of neurodivergence, and sensory processing differences. To our knowledge, there is no empirical evidence looking at treatment outcomes in this population. Future research should adopt an intersectional approach and co-produce research with Autistic TGD people to ensure it is relevant, inclusive, and ethical.

## Data Availability

No datasets were generated or analysed during the current study.

## Glossary

Trans and gender diverse (TGD): TGD is an umbrella descriptor for people whose gender is incongruent with their sex presumed at birth, including trans men, trans women, nonbinary, agender and genderqueer people, as well as those with traditional or Indigenous marginalised genders. Non-TGD people are described as cisgender
Gender dysphoria: Gender dysphoria describes the distress, discomfort or disconnect that a TGD person may (or may not) feel at the incongruence between their gender and gendered body and their presumed sex at birth
Minority stress: Minority stress describes the specific stress minority groups face in hostile social environments, which has a cumulative impact and leads to poorer mental health outcomes in that group
Autistic: Autistic describes a neurotype, with Autistic people sharing a pattern of thinking, moving, sensing and communicating which is divergent from neurotypical standards. Non-Autistic people are described as allistic
Autism Spectrum Disorder (ASD): ASD is the diagnostic label given to Autistic people. The diagnosis requires the Autistic person to show, from early childhood, differences in social communication and social interaction, and repetitive patterns of behaviour, interests and activities, in a way that causes significant impairment
Intersectionality: Intersectionality theory argues that social identity categories overlap and intersect to create unique experiences of marginalisation and privilege
